# No effect of a dopaminergic modulation fMRI task by amisulpride and L-DOPA on reward anticipation in healthy volunteers

**DOI:** 10.1007/s00213-020-05693-8

**Published:** 2020-11-02

**Authors:** Oliver Grimm, Magdalena Nägele, Lea Küpper-Tetzel, Moritz de Greck, Michael Plichta, Andreas Reif

**Affiliations:** Department of Psychiatry, Psychosomatic Medicine and Psychotherapy, University Hospital, Goethe University Frankfurt am Main, Frankfurt, Germany

**Keywords:** Reward, Dopamine, Monetary incentive delay, Striatum, L-DOPA, Amisulpride

## Abstract

**Rationale:**

Dysregulation of dopaminergic neurotransmission, specifically altered reward processing assessed via the reward anticipation in the MID task, plays a central role in the etiopathogenesis of neuropsychiatric disorders.

**Objectives:**

We hypothesized to find a difference in the activity level of the reward system (measured by the proxy reward anticipation) under drug administration versus placebo, in that amisulpride reduces, and L-DOPA enhances, its activity.

**Methods:**

We studied the influence of dopamine agonist L-DOPA and the antagonist amisulpride on the reward system using functional magnetic resonance imaging (fMRI) during a monetary incentive delay (MID) task in *n* = 45 healthy volunteers in a randomized, blinded, cross-over study.

**Results:**

The MID paradigm elicits strong activation in reward-dependent structures (such as ventral striatum, putamen, caudate, anterior insula) during reward anticipation. The placebo effect demonstrated the expected significant blood oxygen level–dependent activity in reward-dependent brain regions. Neither amisulpride nor L-DOPA led to significant changes in comparison with the placebo condition. This was true for whole-brain analysis as well as analysis of a pre-defined nucleus accumbens region-of-interest mask.

**Conclusion:**

The present results cast doubt on the sensitivity of reward anticipation contrast in the MID task for assessing dopamine-specific changes in healthy volunteers by pharmaco-fMRI. While our task was not well-suited for detailed analysis of the outcome phase, we provide reasonable arguments that the lack of effect in the anticipation phase is not due to an inefficient task but points to unexpected behavior of the reward system during pharmacological challenge. Group differences of reward anticipation should therefore not be seen as simple representatives of dopaminergic states.

## Introduction

Rewards are crucial things in life: whether we strive for food, sex, social signals, or illicit drugs, we experience features of our sensory perception as more salient than others and approach towards them. Dopamine is an evolutionarily conserved neurotransmitter. In model organisms like zebrafish, dopamine modulates exploitative and exploratory behavior. Dopamine is involved in neuronal reward signals where it represents and processes crucial aspects of reward functions. However, this so-called reward system is a heterogeneous concept used in today’s neuroscience to capture a variety of different neurochemical, anatomical, neuropsychological, and clinical concepts. While the anatomic definition concentrates on parts of the limbic system with core features like connectivity to the ventral striatum and the dopaminergic midbrain nuclei, the neuropsychological definition tries to define unifying features like reward-driven associative learning. This is the basis of the learning process in operant conditioning (Schultz 2015a): the animal or the participant receives a reward upon reaction to a conditioned stimulus. This stimulus is thought to be mainly independent of the reward’s valence but is as part of a learning scheme associated with the reward’s value. It can be altered by drugs and different neurological or psychiatric conditions. Dysregulation in the reward system has been discussed in disorders ranging from schizophrenia to attention-deficit hyperactivity disorder (ADHD) and has been most often interpreted as a dysfunction of the dopaminergic neurotransmission (Der-Avakian et al. 2016).

A popular and well-validated approach in neuroimaging is to use the monetary incentive delay (MID) paradigm. Its suitability for neuroimaging was demonstrated almost 20 years ago (Knutson et al. 2001; Kirsch et al. 2003). Via echo planar imaging (EPI) neuroimaging, the blood oxygen level–dependent (BOLD) response to a conditioned stimulus during the anticipation phase is measured. The task has been used in several variants which can lead to different effects depending on task methodology like contingency rate of the reward (Plichta and Scheres 2015). This MID task has been demonstrated to have good test reliability (Plichta et al. 2012). In a combined PET-raclopride-fMRI study, the anticipation phase was linked to a surge in dopamine (Schott et al. 2008). Other studies indicated sensitivity to both a phenylalanine depletion (Bjork et al. 2014), a dopamine precursor, and to amphetamine, a dopamine reuptake inhibitor (Knutson et al. 2004). Therefore, several findings in psychiatric patients, like a blunted BOLD response during anticipation in patients with ADHD (Plichta and Scheres 2014) or schizophrenia as well as first-degree relatives (Grimm et al. 2012, 2014), were interpreted in the context of dopaminergic theories.

Most variants of the MID have concentrated on the anticipatory phase and contrasted a conditioned stimulus (CS+) with a control stimulus (Oldham et al. 2018). Several disorders show a blunted response to the CS+ (Hägele et al. 2015). In animals, the conditioning is a learning process represented by a transfer of dopaminergic reactivity from the moment of the reward to the anticipation of the reward represented by the CS+ (Schultz 2015b). A stronger reaction to the reward might indicate a reward learning deficit; therefore, the difference between anticipation and reward feedback offers additional information.

As several studies interpret the reaction pattern of the MID task as corresponding to changes in dopaminergic regulation, we wanted to test whether dopaminergic drugs can elicit changes in neuronal responses. While several previous studies used between-group designs (cf (Martins et al. 2017) ), it is preferable to use a within-subject design for a one-time pharmacological challenge as inter-brain variability is much higher than the effect to be observed.

The MID task has been reported with several different dopaminergic challenges, but results remain contradictory. Ye and colleagues (Ye et al. 2011) observed an increase of nucleus accumbens activation during reward anticipation after intake of the dopamine agonist pramipexole. A small study in *n* = 8 participants saw equalization of gain and loss (Knutson et al. 2004), whereas in rodents a dose-dependent decrease or increase of local BOLD activity was demonstrated with fMRI for different amphetamine doses (Ren et al. 2009). Some previous and older studies might suffer from methodological problems, e.g., a small number of participants, neuroimaging statistics prone to type I errors.

We choose the dopamine precursor L-DOPA as a dopaminergic compound and contrasted it not only to placebo but also to the dopamine antagonist amisulpride, which is one of the most selective D2 blockers available in humans.

L-DOPA is the immediate precursor of dopamine in a reaction catalyzed by DOPA decarboxylase, a rate-limiting step enzyme involved in dopamine synthesis (Lewitt 2015). Once having passed into the brain, L-DOPA becomes available for decarboxylation within dopaminergic fibers and terminals and, thus, is thought to increase dopamine synthesis and release in target areas in demand. Two hundred fifty milligrams of L-DOPA doubles the ventricular CSF levels of the dopamine metabolite DOPAC (Raftopoulos et al. 1996) and increases striatal dopamine synthesis (measured by PET) in healthy subjects (Black et al. 2015), with minimal effects on tonic dopamine release at rest (Floel et al. 2008).

The understanding of dopamine’s acute function has far-reaching implications as it not only enables the understanding of psychopharmacological drugs and their mechanism but also may serve as a model for a variety of neuropsychiatric disorders that show dopaminergic deficits.

### Objectives

Pharmacological manipulation of human reward processing is still scarce and faces several methodological and logistic challenges. Nevertheless, they can offer translational insights and analogies to animal models and help to evaluate mechanistic models. Therefore, our objective was to test dopaminergic signaling by evaluating a pharmacologic challenge. Our hypothesis was (1) that both drugs should modulate the anticipatory phase in its most salient part (contrast win > control) and (2) that amisulpride leads to a blunted striatal signal while L-DOPA would boost the anticipatory response.

## Material and methods

### Participants

The study includes 45 mentally and physically healthy subjects (22 male, age 22.81 years SD 2.71). Recruitment took place at the Goethe University Frankfurt am Main and social networks.

Inclusion criteria were age between 18 and 50 years. Exclusion criteria were intolerance to the study drugs, mental illness, serious acute or chronic physical diseases, and pregnancy, and exclusion criteria is MRI examination. Participants were examined by a registered psychiatrist.

The project was carried out in accordance with the provisions of the Declaration of Helsinki (World Medical Association 2013) and the European guidelines on Good Clinical Practice and was approved by the Ethics Committee of the Medical Faculty of the J.W. Goethe University Frankfurt am Main (reg. no. 256/16). The study was registered in the German register of clinical studies on November 11, 2016, with the study ID DRKS00011209.56. Subjects gave written informed consent. The subjects received 10€ per hour for participation. In addition, the monetary gain of the Monetary Incentive Delay Task of all three measurement dates was paid out to the volunteers.

### Monetary incentive delay fMRI paradigm

**Fig. 1:**
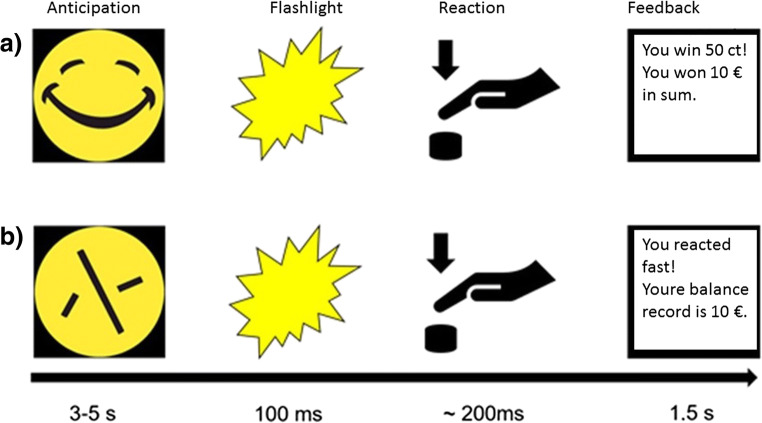
Monetary incentive delay task: event-related functional magnetic resonance imaging (MRI): 60 trials, 30 of which are winning conditions and 30 control conditions. In the anticipation phase, a smiley (**a**) or a control circle (**b**) is shown. After a flash is displayed, the participant must react as quickly as possible by pressing a button, followed by a feedback: (a) winning condition: feedback by winning money, 50 cents or 2 € booster and (b) control condition: written feedback about the reaction’s success

We used an MID task in combination with fMRI measurement. The task was validated in previous studies (Plichta et al. 2012; Boecker et al. 2014; Grimm et al. 2014) and proved that this fast event–related paradigm works well for repetition time (TR) between 2 and 2.5 s. By using randomly jittered intervals, it is possible to detect the BOLD signal course despite a long TR, as shown by independent research groups (Lamm et al. 2014). Volunteers were shown 30 “smileys” as well as 30 neutral, scrambled “control smileys” on a screen in the MRI in an unpredictable order, to which they had to react as quickly as possible with the push of a button after a flashlight occurred. After presentation of the smileys (or conditioned stimuli), the participants earned a monetary feedback of 50 cents if they reacted quickly. In order to prevent habituation, participants were unexpectedly rewarded with a booster prize (max. four times) of 2 € in between (Riba et al. 2008; Plichta et al. 2013). If the reaction time was too slow, the participant did not win money. The smiley represents the winning condition (a). In a control condition (b), the test persons were shown a scrambled smiley, a yellow circle (see Fig. 1). In the control condition only, a written positive or negative feedback was provided. The money won, as well as the current account balance, was displayed on the screen after each run (e.g. “You win 50 cents, the account balance is 10 euros”).

After each trial, the reaction time was adapted by 10% depending on success (tougher next trial) or miss (easier next trial). In order to increase the expectation of rewards, the participants were informed beforehand that the money won in the MID task will be paid out in cash directly after the measurement.

### Pharmacological challenge

The measurements were performed at the Brain Imaging Center (BIC) in Frankfurt am Main as a placebo-controlled, double-blind cross-over study. In total, the volunteers were scheduled for three consecutive appointments at intervals of 1 week each (at least 4 days). This minimum interval between the fMRT measurements was chosen to ensure that at the time of the measurement, drugs from a previous measurement were eliminated from the body of the participants. The plasma half-life (*t*1/2) is *t*1/2 = 1–1.5 h for L-DOPA and *t*1/2 = 17 h for amisulpride. After five half-lives, it can be assumed that a drug has been sufficiently eliminated from the body and therefore cannot influence the subsequent measurement.

At each session, the subjects received placebo, 125 mg L-DOPA, or 200 mg amisulpride. Each study participant hence received each of the medications exactly once in randomized order.

The placebo tablet used consisted of lactose powder packaged in gelatin capsules. The same gelatin capsules were used to package the other pharmaceuticals to ensure that neither the subject nor the investigator could tell visually which tablet was being used. The L-DOPA tablet used was a composition of 100 mg L-DOPA and 25 mg carbidopa, an aromatic L-amino acid decarboxylase that catalyzes the conversion of L-DOPA to dopamine. In contrast to L-DOPA, carbidopa does not cross the blood-brain barrier and thus only acts on the periphery of the body. Thus, the central availability of L-DOPA can be increased, since in uninhibited dopadecarboxylase 95% of L-DOPA would be decarboxylated in the periphery and thus would not be effective in the CNS (Muthuraman et al. 2018).

The unblinding did not take place until the MRT analysis of all volunteers had been completed. Both before and after each measurement, pulse per minute and blood pressure were measured in each subject. The study participants received the study medication as oral application approximately 90 min before the start of the fMRI measurement. The waiting time between taking the medication and starting the resting state measurement is based on the time (tmax) until the maximum plasma concentration is reached. This is indicated with tmax = 0.5–1 h, for L-DOPA (Contin and Martinelli 2010), and with amisulpride a first plasma concentration peak is also observed one hour after oral intake (Rosenzweig et al. 2002). For safety reasons, the subjects were monitored by a breathing belt and an oxygen saturation clip during MRI. After each fMRI session, a 60-min follow-up period was scheduled to detect any side effects of the medication.

### MRI measurement

The functional MRI data were collected with a SIEMENS MAGNETOM Trio syngo MR A35 at the Brain Imaging Center of the Goethe University Frankfurt am Main. The functional scans were recorded with a 3-T scanner and an 8-channel head coil. Foam pads were used to fixate the participants; head and to minimize movement of the head. An anatomical sequence (MPRAGE sequence, magnetization prepared rapid acquisition gradient echo) was followed by the functional image data of the MID task recorded with a gradient echo EPI sequence (echo planar imaging images). The MID task consisted of 60 experiments in an event-based design, including 30 monetary win condition trials and 30 control trials. The anatomical T1 scan was recorded with a slice thickness of 1 mm, a repetition time TR = 1900 ms, an echo time TE = 3.04 ms, and a voxel size of 1 × 1 ×1 mm^3^. The EPI recordings during the MID task were made layer by layer with a layer thickness of 2.5 mm, a repetition time TR = 2500 ms, and an echo time TE = 30 ms. The size of a voxel was 3 × 3 × 2.5 mm^3^. We used a flip angle of 90°, and a field of view of 192 mm with 100% phase. The phase-encoding direction was from anterior to posterior. The behavioral data collected at the same time were recorded in the form of reaction times (corresponding to the measured time from flashlight to pressing a button).

### Analysis of behavioral data

Behavioral data analysis was done with SPSS Version 27. The main dependent variable was the condition-specific reaction time. We tested for normal distribution, followed by paired *t* tests and a non-parametric test for paired samples. We tested the differences between L-DOPA and placebo, amisulpride and placebo, and amisulpride versus L-DOPA.

### Analysis of movement

Movement is a major source of artifacts in fMRI research. As both L-DOPA and amisulpride have clinical effects on movement, we wanted to test for effects on movement during scanning. We calculated for each participant the mean movement as frame-wise displacement (FD) for the L-DOPA, amisulpride, and placebo conditions. FD was calculated with the six regressors derived from the realignment preprocessing step according to Power et al. (2012): FD_*i*_ = |Δ*d*_*ix*_|+|Δ*d*_*iy*_|+|Δ*d*_*iz*_|+|Δ*α*_*i*_|+|Δ*β*_*i*_|+|Δ*γ*_*i*_| and |∆*d*_*ix*_|=*d*_(*i*−1)*x*_−*d*_*ix*_. We tested for normal distribution of the FD values with the Kolmogorov-Smirnov test and used a signed Wilcoxon-rank test where appropriate.

### fMRI data preprocessing

The fMRI data analysis made use of statistical parametric mapping (SPM) (Flandin and Friston 2008), version SPM12.

Data preprocessing was done in SPM12. EPI images were realigned, slice time corrected, co-registered with T1 MPRAGE images, spatially normalized to standard stereotactic space (Montreal Neurological Institute (MNI) template), resampled to 3-mm isotropic voxels, and smoothed with 8-mm full width at half maximum (FWHM) Gaussian kernel.

### fMRI first-level statistics

In a first-level analysis step, we derived an individual task model for each participant. Based on SPM12, we used a general linear model to derive a voxel-wise hemodynamic response. The task was modeled by including a six-regressor regressor: (1) a win-anticipation condition, (2) a control-anticipation condition, (2) the unconditioned stimulus (flashlight), (3) a monetary feedback for success, (4) a monetary feedback for failure, (5) a non-monetary feedback for success, and (6) a non-monetary feedback for failure. These parts of the paradigm were modeled as zero events (delta functions) and convolved with a standard hemodynamic-response function. The task reaction parameter, which describes the onsets of the flashlight, and six realignment motion parameters (3 translations/rotations) were included as nuisance covariates, removing flashlight- and movement-related signal changes that might be correlated with the experimental design. A high-pass filter with a cutoff frequency of 1/128 Hz was used to attenuate low-frequency components. All analyses were corrected for serially correlated errors by fitting a first-order autoregressive process (AR[1]) to the error term. We calculated the contrast win anticipation > control anticipation, which is the most common used contrast to analyze anticipation in the MID task (Oldham et al. 2018). This contrast was used for the subsequent second-level analysis.

### fMRI-group analysis

To demonstrate the validity of our approach, we calculated a one-sample *t* test for both contrasts. This should demonstrate that our task setup is indeed able to capture the reward system’s response to our task’s events.

Our main analysis uses paired *t* tests to calculate the difference between L-DOPA and placebo, amisulpride and placebo, and L-DOPA and amisulpride. Voxel thresholding for cluster analysis was done with *p* < 0.001 and corrected for multiple comparisons by a clusterwise familywise error (FWE) test (alpha set to *p* < 0.05). As we had strong a priori hypothesis about the involvement of the striatum as center of the reward system, we made use of a small-volume correction, by limiting the analysis to the voxels included in an AAL-atlas mask of the bilateral striatum.

## Results

### Behavioral data

#### Anticipation of monetary reward

The Kolmogorov-Smirnov test indicated a normal distribution for the mean trial type–specific reaction times for each condition placebo, amisulpride and L-DOPA (*p* > 0.05). The paired *t* test did not demonstrate significant differences of reaction times between L-DOPA (*M* = 176.21 ms, SD = 14.93 ms), placebo (*M* = 175.79 ms, SD = 17.08 ms), and amisulpride (*M* = 179.23 ms, SD = 20.36 ms). Neither L-DOPA versus placebo (*t* = 0.18, *p* = 0.86, df = 44) nor amisulpride versus placebo (*t* = 1.44, *p* = 0.16, df = 44) showed a significant influence on reaction times during the win condition of the MID task.

##### Anticipation of non-monetary feedback

Reaction times during amisulpride and L-DOPA were not normally distributed (Kolmogorov-Smirnov test, *p* < 0.05). Analysis was done with the Wilcoxon sign-rank test. Reaction times during L-DOPA (*M* = 190.43 ms, SD = 19.75 ms) and placebo (*M* = 189.61 ms, SD = 21.34 ms) as well as between amisulpride (*M* = 192.73 ms, SD = 25.06 ms) and placebo did not differ significantly (*p* > 0.34, *z* < − 0.48, *n* = 45).

### Movement

We defined a threshold of movement > 3 mm as exclusion criterion. No participant had to be excluded. The Kolmogorov-Smirnov test indicated a non-normal distribution (*p* < 0.05) therefore the Wilcoxon signed-rank test was applied. The mean changes in movement (measured as framewise displacement, FD) were during the L-DOPA condition (*M* = 0.22 mm, SD = 0.11 mm) compared with placebo (*M* = 0.20 mm, SD = 0.10 mm) resulting in a non-significant difference (*p* = 0.53, *z* = − 0.64). The FD for amisulpride (*M* = 0.22 mm, SD = 0.12 mm) and placebo also did not differ significantly (*p* = 0.25, *z* = − 1.15).

### Analysis of the MID-fMRI data

#### Main effect of the MID task

**Fig. 2:**
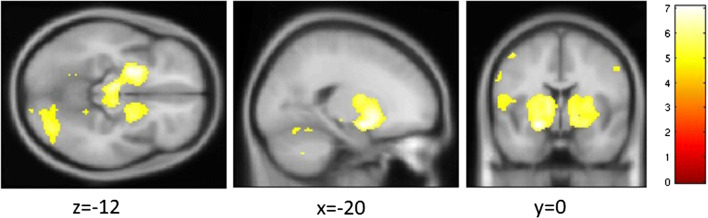
Main effect of task: contrast anticipation of monetary reward > anticipation of control feedback. For illustrational purposes, voxels below pFWE < 0.05 are color coded depending on the effect size (color bar to the right indicates the *T* value)

We summarize the effect of contrast reward anticipation versus control anticipation (Fig. 2; Table 1). There, we demonstrate the main effect in the placebo condition to validate our claim about the underlying neuroanatomy. Results are shown as result of a one-sample *t* test.

**Table 1:** Main effect for the contrast monetary reward anticipation > control anticipation

Brain region of cluster’s peak	Cluster location (MNI)	Cluster size (*k* in voxel)	Anatomical cluster content (*k* in voxel)	Cluster pFWE	*T* statistics
Occipital lobe R	34 − 52 − 20	1881	919 right cerebellum 672 occipital lobe 376 fusiform gyrus 295 lingual gyrus 235 temporal lobe	< 0.001	7.07
Putamen R	− 20 0 − 12	6781	1225 putamen 741 thalamus 638 midbrain 525 inferior frontal gyrus 493 insula 274 precentral gyrus 258 temporal lobe	< 0.001	7.06
Cerebellum L	− 38 − 52 − 26	876	622 left cerebellum 191 fusiform gyrus 139 occipital lobe	0.005	6.31

Montreal neurological space (MNI) corresponds to the *x*-*y*-*z* coordinates in millimeters in the MNI template. Cluster size is given in *k* voxel. Effect size T. Significance cluster pFWE with cluster-defining threshold *p*-uncorrected < 0.001. Anatomical cluster content indicates size in *k* voxel for the largest regions with *k* > 100

*L* left, *R* right

The main effect of the contrast monetary anticipation > non-monetary anticipation is shown in Fig. 2 and Table 1. The main effect can be summarized as spanning regions from the putamen, especially the ventral striatum, thalamus, midbrain, the inferior frontal gyrus, the fusiform gyrus, and parts of the cerebellum.

#### Effect of the pharmacological challenge

Results were considered significant when a cluster threshold of *p* < 0.001 (uncorrected) met an alpha of *p* < 0.05 (multiple-testing comparison via family-wise error). We calculated a directed paired *t* test looking for the contrast monetary versus control reward anticipation and compared the sessions placebo versus amisulpride, placebo versus L-DOPA, and L-DOPA versus amisulpride. None of these contrasts gave significant voxels for whole-brain testing (pFWE > 0.7). In the case of the L-DOPA versus amisulpride comparison, there were no detectable suprathreshold clusters. As we considered the nucleus accumbens an important a priori region of interest, we applied a small volume correction to avoid type II errors. This masked analysis failed to detect significant voxels at our cluster threshold (*p* > 0.001).

## Discussion

### Summary and discussion of specific results

We here used pharmacological modulation of the dopamine system to probe the reward system in a monetary incentive delay task. Contrary to our expectation, we were neither on the behavioral nor on the neural level able to find an effect of L-DOPA or amisulpride on the two contrasts studied. This has both methodological implications and implications for studies using the MID task, as the latter is often interpreted within a dopaminergic framework.

We focused our analysis on the contrast reward vs. control anticipation as this is the most studied contrast in MID tasks (Oldham et al. 2018) and has been evaluated in several clinical populations.

Previous studies identified this reward anticipation contrast as a potent activator of the striatal system or—in a broader context—the reward system. We were able to replicate the well-known activation pattern including increased BOLD response in the ventral striatum, insula, thalamus, and cingulate cortex in our main effect analysis of the placebo condition (but also the two drug conditions).

A very small previous study on eight adults found a blunting of activity in the ventral striatum and an equalization of response to gain and loss during amphetamine. This was interpreted as an enhancement of tonic over phasic activity during reward anticipation (Knutson et al. 2004). While this study was underpowered, it nevertheless was the first to suggest a blunting of the reward anticipation by a dopaminergic agonist, assuming an inverted U curve of dopamine effects, mediated via a shift in the phasic signal-to-noise ratio.

### Limitations

A certain limitation of our study is the fact that we did not use a specific D1- or D2-receptor agonist but a precursor of the dopamine synthesis pathway. However, L-DOPA (L-DOPA) is catalyzed in a rate-limiting step by the DOPA decarboxylase, a bottleneck of dopamine synthesis (Lewitt 2015). A direct infusion into the brain leads to a marked increase in dopamine synthesis. The administration of 250 mg L-DOPA leads to a burst of a dopamine metabolite pointing to a drastic increase of synthesis (Raftopoulos et al. 1996). Therefore, L-DOPA provides a fast-acting dopaminergic boost as also demonstrated by its fast clinical onset and impulsive-behavior in Parkinson’s disease patients (Castrioto et al. 2013).

Amisulpride has been used in previous pharmacological challenge studies (cf review Martins et al. 2017). The simple interpretation that amisulpride was given in not sufficient dose can be partly countered by referring to behavioral, endocrinological, and neuroimaging studies which successfully used 200 mg amisulpride. Nevertheless, amisulpride has some paradoxical dose-dependent features: Presynaptic autoreceptors lead to an increase of dopamine, and it is likely that at lower amisulpride dosing, the dopamine-facilitating effect will dominate. However, the exact cutoff is not well-known. Modulation of local resting-state fMRI activity was recently reported in a small study (Metzger et al. 2015), which showed an effect of plasma amisulpride level (with 200 mg oral intake) on putamen functional connectivity to the dopaminergic midbrain. The authors thought this suggests a pro-dopaminergic effect. Contrary to this interpretation, human SPECT binding suggests a predominant antagonistic effect via a D2 receptor occupancy between 60 and 80% for 200 mg amisulpride (la Fougère et al. 2005). A possible interpretation would be that amisulpride leads e.g. L-DOPA to an enhanced dopaminergic tone. As the BOLD fMRI signal most likely reflects the phasic dopaminergic component, it gets lower even if the global dopamine increases. If we selected amisulpride at a dose where the dopamine-agonistic effects equalized the dopamine-antagonistic effect, we would not be able to demonstrate a BOLD fMRI signal. However, such an effect has so far not been reported.

Apart from presynaptic autoreceptors, a low dopamine receptor occupancy must be discussed, too. In a previous study, 200 mg sulpiride (MA et al. 2008) gave only about 17% D2-receptor occupancy. However, while both are substituted benzamides, lipophilicity and D2/D3-receptor binding are distinct between amisulpride and sulpiride.

Another possibility is that amisulpride and L-DOPA have direct, non-specific effects on the cerebral vasculature because dopamine has some effects on the regulation of vasculature.

A different limitation is originated in our fMRI task design. While our task was optimized for the anticipation phase, it seems that the task was not optimal for the analysis of outcome. Reasons for this include a low number of trials, which did not suffice for optimal adaption of individual task performance, and high correlation between feedback regressors and the target regressor, which is a common issue in all MID tasks and an uneven distribution of omitted trials among participants in the outcome phase, leading to a high variability in contrast to the reward phase. As previous efforts of our group concentrated exclusively on reward anticipation, we cannot make a statement about contrasts comparing reward anticipation and reward feedback or reward feedback hits vs. misses. While these contrasts are not as widely used and validated (Oldham et al. 2018), a limitation of our study is nevertheless the lack of a detailed outcome analysis. We cannot exclude the possibility that some drug effect is hidden in the outcome parts of the MID task. Future MID-based studies should not only study different doses of amisulpride to better understand potential presynaptic effects but use more trials with more fine-grained levels of reward. When reporting negative results, it is important to consider alternative explanations. One obvious alternative explanation, that the reward MID task is not sufficiently activating the reward system, is countered by the large main effect of our task. The task was optimized to produce a large anticipation BOLD fMRI response in the striatum by including an additional “boost” trial (Boecker et al. 2014) as also seen in our neuroimaging data. This might lead to a ceiling effect in that the dopamine system is already at its peak activity and cannot be further activated by exogenous dopamine increases. However, there is no previous evidence for such a ceiling effect and it would also be in contradiction to previous findings (Martins et al. 2017). In a recent paper, we reported on resting-state fMRI effect in this sample and a significant effect of drugs on functional connectivity (FC) (Grimm et al. 2019). However, our seed FC analysis used subcortical and midbrain seeds and was not tested in their ability to predict the activity of the MID task. In summary, we found a strong effect of L-DOPA and amisulpride on FC, especially an increase of dopaminergic midbrain nuclei to the insula in L-DOPA. While this effect does not offer a prediction for the MID task, it demonstrates that drug doses were sufficient to produce detectable neuroimaging effects.

Another alternative explanation points to the insufficient application of the drug: maybe the drug did not reach a sufficient peak concentration in the brain during scanning? While we cannot counter this argument by CSF concentration measurements, we used previously established drug application paradigms in terms of timing and dosing. Our scanning time frame was adapted to the well-known pharmacokinetics of both amisulpride as well as L-DOPA. The L-DOPA tablet used was a composition of 100 mg L-DOPA and 25 mg carbidopa, an aromatic L-amino acid decarboxylase that catalyzes the conversion of L-DOPA to dopamine. In contrast to L-DOPA, carbidopa does not cross the blood-brain barrier and thus only acts on the periphery of the body. Thus, the central availability of L-DOPA can be increased, since otherwise 95% of the L-DOPA would be decarboxylated in the periphery and would not be effective in the CNS (Muthuraman et al. 2018). About 90 min after taking the drug, the MID fMRI measurement began. We based the waiting time between oral intake of the drug and the MID fMRI measurement on the drug’s tmax. The tmax for L-DOPA is 0.5–1 h (Contin and Martinelli 2010), and for amisulpride a first plasma concentration peak is observed within 1 h after oral intake (Rosenzweig et al. 2002). Not all previous studies of neural or behavioral L-DOPA effects demonstrate simple effects on reward: A recent report (Wittmann and D’Esposito 2015) showed that 100 mg L-DOPA did not affect reward processing, neither on the behavioral level nor on the neural level. However, it enhanced striatal activity for punishment cues compared with reward cues. Nevertheless, several studies demonstrated the effects of L-DOPA in doses between 100 and 150 mg (cf (Martins et al. 2017)).

## Conclusion

In summary, we did not find evidence that reward anticipation contrast tested in our MID task is modulated by a challenge of the dopamine system using an antagonist or agonist. This is an unexpected finding because the dopaminergic reward and probabilistic error tracking signal has been identified as a relevant mechanism in a reward-associated operant conditioning task (Schultz 2015a). Besides, a study with an MID-fMRI task demonstrated an increase of ventral striatal activation during reward anticipation for the dopaminergic agonist pramipexole (Ye et al. 2011). However, these studies are extremely heterogeneous in terms of paradigms and methods (cf (Martins et al. 2017)). We do not prove that there is no drug effect at all (cf Grimm et al. 2019) but state this only for the reward anticipation contrast. Nevertheless, as this contrast is one of the best-evaluated reward fMRI tasks in neuropsychiatry and linked tight to the phasic dopamine response (Schott et al. 2008), we believe that this negative effect bears important information for both clinicians and basic researchers.

A large body of data gathered through human neuroimaging studies demonstrates the activation of reward-related neuroanatomical structures by reward-related stimuli and reward-related actions. It is plausible that the non-invasive fMRI BOLD signal in the striatum resembles several aspects of the reward prediction error found in neurophysiologic experiments (Schultz 2015a). However, our study casts some doubt on the next step in the argumentation chain, namely, that striatal BOLD fMRI likely reflects the dopamine prediction error signal, as it is not influenced by the dopamine-modulating agents in our study. We therefore propose that the exact mechanisms linking dopamine and neurovascular coupling are more complex. While our interpretation cannot provide a new and complete model, we nevertheless believe that the falsification of a theory is a scientific cornerstone (Popper 1963) and opens the perspective for new scientific questions. Finally, our study is more definitive than previous ones given that it has much higher power to detect negative findings with *n* = 45 participants in a crossover trial, than previous, much smaller samples. In a review that collected sample size of pharmaco-fMRI studies of reward-associated paradigms in 61 studies, only one study had a larger sample size in a within-subject cross over design (Martins et al. 2017).

While we believe that pharmacological manipulation of the dopaminergic system is an invaluable tool to study cause–effect-based models of reward, the reward anticipation MID task does not seem to be change-sensitive to pharmacological challenges. Blunted reward anticipation has been reported in several clinical populations (Hägele et al. 2015). It has been often linked directly to a change in dopaminergic neurotransmission. However, interpretation of findings in the clinical population should be done with caution as our study indicates that blunted reward anticipation does not necessarily point to a direct dysregulation of dopaminergic neurotransmission.

This also has implications for the design of further studies. Following an RDoC approach and more direct bench-to-bedside thinking, it has been discussed whether fMRI techniques can be applied as biomarkers in drug development (Schwarz et al. 2011). Such an application will only make sense if fMRI assays (like the MID task) deliver robust, reliable, and validated results. While retest reliability is good, this does not necessarily point to reward anticipation as a sensitive imaging biomarker for clinical studies, as our study casts doubt on the use of the reward anticipation contrast as an assay for the evaluation of dopaminergic functions. Future (f)MRI studies might look into the effects of dopaminergic challenges in other paradigms and techniques, e.g., spectroscopy which might yield additional biochemical data and use the specific recommendations discussed above.
